# The Welfare of Dairy Cows in Pasture, Free Stall, and Compost Barn Management Systems in a Brazilian Subtropical Region

**DOI:** 10.3390/ani12172215

**Published:** 2022-08-28

**Authors:** Paula de Andrade Kogima, Taciana Aparecida Diesel, Frederico Márcio Correa Vieira, Ana Luiza Bachmann Schogor, Alana Aparecida Volpini, Géssica Jaine Veloso, Patrícia Ferreira Ponciano Ferraz, Maria Luísa Appendino Nunes Zotti

**Affiliations:** 1 Department of Animal Science, Santa Catarina State University (UDESC), Florianópolis 89815-630, Brazil; 2 Department Animal Science Department, Federal Institute of Education, Science and Technology of Maranhão (IFMA), São Luís 65075-441, Brazil; 3 Department of Animal Science, Federal Technological University of Paraná (UTFPR), Boa Esperança 85660-000, Brazil; 4 Department of Agricultural Engineering, Federal University of Lavras (UFLA), Lavras 37200-900, Brazil

**Keywords:** welfare quality protocol, water points, lameness

## Abstract

**Simple Summary:**

Understanding the critical points of welfare of animal production systems is an important tool to identify strategies to improve the quality of life of animals. This work showed that rearing systems affect the welfare of dairy cows when the Welfare Quality^®^ protocol was applied in 51 farms; 17 in the pasture-based, 17 in the compost barn and 17 in the free stall systems. The results showed that the pasture-based system scored better in most measures, except for those related to the provision of quality water to the animals. The free stall system has more welfare issues than the pasture-based system and the compost barn system. The compost barn system has advantages in the “good housing” principle, when compared to the free stall. This work carried out a survey that can be used as a basis for the establishment of strategies to improve animal welfare, in a specific way to the adopted rearing system.

**Abstract:**

The effect of milk production systems on the welfare of dairy cows has been studied worldwide, but studies that compare pasture-based, compost barn, and free stall systems, according to animal welfare, are more scarce. In this work, the welfare of 51 dairy herds, including 17 from each management system, was investigated through the application of the Welfare Quality^®^ protocol. Descriptive statistics and the Kruskal–Wallis non-parametric method were used to analyze variables. In the present work, the welfare of the evaluated herds was significantly better in the pasture-based system than in the confinement systems. However, the pasture-based system presented weaknesses in providing water resources. The compost barn had fewer animal welfare critical points than the free stall system, as well as it was better than the free stall in measures related to comfort and health. The free stall did not present better scores than the other systems. It is concluded that the welfare of dairy cows is affected by the rearing system, with better scores, in most measures, in the pasture-based system, followed by the compost barn and, finally, the free stall.

## 1. Introduction

The moral concerns of science in relation to animal welfare (AW) were potentiated after the publication of Ruth Harrison’s book, entitled *Animal Machines* (1964), causing a great effect on society and impelling the government of the United Kingdom to organize the so-called Codes of Practice [1] (pp. 218–221). The Codes of Practice were the way the English government regulated measures that would guarantee a certain degree of welfare to production animals.

In view of societal changes in the treatment of animals, the Farm Animal Welfare Council proposed the so-called five AW freedoms, which are principles used for AW assessments, regulating what is acceptable or not, taking into account respect for the physical and mental state of animals, which implies good health and a sense of well-being [2]. This concept should be used in the evaluation of different environments, such as farms, slaughterhouses, commercial facilities, and vehicles that transport live loads [3].

The restrictions that dairy cattle production systems can bring to the expression of the natural living of cows have led to growing concerns on the part of society, and these restrictions are prevalent in confinement systems [4,5] (pp. 251–270). Natural living (or telos) was defined by Aristoteles as essential functions (locomotion, reproduction, sensation, nutrition, etc.) that must be fulfilled because they are part of the natural constitution of each animal species [6]. In contemporary times, Rollin [6] considered that AW can be defined in terms of meeting natural living, that is, meeting different interests and needs that affect the biological and psychological nature of animals.

Three aspects in the evaluation of AW gain emphasis: the manifestation of the normal repertoire of behaviors of the species in question; the emotional state, which would be the avoidance of suffering and the possibility of satisfaction; and biological functioning, related to health, reproduction, and growth [7]. Realizing the different approaches that researchers prioritized in the evaluation of AW, Fraser [8] carried out a study that considered three main components that constituted three interconnected spheres for the adequate evaluation of AW: basic health and adequate biological functioning (with evidence in the absence of disease and injury); affective states (positive and negative); and a natural life, also called naturalness. Fraser [8] showed that none of these three components should be more important than the other, but the AW assessment should use them together.

In 2009, a group of scientists published the Welfare Quality^®^ protocol (WQ) [9], the result of an interdisciplinary project (2004–2009) that aimed to formulate a protocol for evaluating the welfare of farm animals, that took into account the expectations of consumers, producers, and traders and that, preferably, used measures based on animals [10]. This is an instrument that more objectively assesses the welfare of farm animals, such as dairy herds.

In 2019, Brazil was considered the fourth-largest milk producer globally, with 25.1 billion liters produced in the year, and Santa Catarina (SC) was considered the fifth-largest inspected milk producer in the country [11]. The predominant dairy cattle system in Brazil is the pasture system, followed by the free stall (FS) confinement and compost barn (CB) models.

Given the importance of Brazil in world dairy production and the recent migration from pasture-based systems to confinement, further studies are needed to assess the welfare of dairy herds. A study carried out in 2013, in the western region of SC, Brazil, in which family dairy farms were evaluated in extensive, semi-intensive, and pasture-based (PT) systems, showed that clinical manifestations such as lameness and hock lesions were predominant in semi-intensive dairy farms [12]. In this study, cows with low body scores and inappropriate milking infrastructures had a higher prevalence in the extensive and PT systems. In addition, specific problems were observed in the three types of systems: tick infestations, subclinical mastitis, poor hygiene during milking, and deficiencies in the availability of drinking water and in the shaded areas [12].

Recently the emergence and growth of the CB system has been observed, a still rare system in Brazil, at the time of the mentioned work. The work developed by Radavelli et al. [13] characterized the CB system in the west region of SC, indicating the massive presence of this system in the region in early 2017. In this sense, work is needed to show the impact of the migration from PT production systems to FS and CB on the welfare of dairy cows as it has already been shown that confinement limit the manifestation of the natural behaviors [14].

The WQ protocol is used worldwide to assess the welfare of dairy herds in confinement systems, but proposals have been made to adapt the application of this instrument to extensive and PT systems [15,16]. Our objective with this work was to verify how different systems of dairy cows affect the AW. For this purpose, the critical points of AW of the PT, FS, and CB systems were studied, based on the principles, criteria, and measures of the WQ protocol.

## 2. Materials and Methods

### 2.1. Evaluated Places, Farms and Animals

The evaluation of the welfare of dairy cows was carried out through the application of the WQ protocol in 51 farms, being 17 PT, 17 CB, and 17 FS systems, distributed in 21 cities in the western region of the state of SC, Brazil, which is characterized by the humid subtropical climate, with precipitation of up to 2000 mm/year [17]. In addition to these, 6 other farms were visited for training on the application of the protocol, but they were not part of the sample. All farms were evaluated by a single evaluator who participated of the training and the others formed a discussion group. The training group was important for learning and adequacy of details in the assessment of dairy cow welfare.

For the classification of a production system as PT, we considered characteristics presented by Costa et al. [12], i.e., the intensive production of pastures, with frequent use of fertilization of the field and rotation of paddocks, and the supplementary feeding of the cows in the feeders. All farms in this group performed 2 milkings a day. On the other hand, confinement systems were characterized by the permanence of cows in facilities without free access to the external area, with water points available inside the housing and food based on commercial concentrates [15] forage and silage. The FS and CB farms were milked 2 to 3 times a day. All the farms evaluated used the mechanical milking system, were located up to 250 km from Chapecó (SC), had the systems established for more than 6 months, and did not undergo renovations in the facilities.

In an FS system, cows do not have much room to move freely [18], as they are in a restricted area divided into a space for feeding and another for resting, with individual beds arranged side by side and lined with crushed rubber or sand, for example [19]. The CB system emerged from adaptations made to the loose housing system [20]. In the loose housing system, cows are kept in fenced and covered places for collective rest, thus providing shelter [19]. In addition, cows have more freedom to move and present the natural behaviors of the species [21] than in the FS system.

Visits to the farms were carried out from September 2019 to August 2020, starting between 4:00 am and 8:00 am, always at the first milking and ending between 11:00 am and 14:00 pm. We evaluated 2770 lactating cows (1394, 893, and 483 cows in FS, CB, and PT, respectively). The average number of lactating cows (followed by the standard deviation) in the farms was 82 ± 57.0, 52 ± 23.53, and 28 ± 15.52 for FS, CB, and PT, respectively. The largest herd evaluated had 221 lactating cows (FS), while the smallest had 8 cows (PT). The average milk production (followed by the standard deviation) of the last 3 months before the visit was 24.8 ± 7.1 L/cow.day (29.14 ± 3.3; 26.4 ± 6.5; and 19.1 ± 6.8 L/cow.day for FS, CB, and PT, respectively).

For the qualitative evaluation of the herd and the behavioral observations, the total number of lactating cows in each farm was evaluated. For the avoidance distance test and the clinical evaluation, we used a sample proposed by the WQ protocol [9]. The total sample number of cows evaluated was 1523 (601, 519, and 403 from FS, CB, and PT, respectively).

### 2.2. Adaptations to the Evaluation of the Welfare Quality^®^ Protocol

Measurement evaluation was performed according to the order proposed by the WQ protocol, except for the lameness measure. The protocol recommends that the score of lameness be visualized when the cows are walking on a non-slip surface, on a straight line, and on a hard and level surface [9]; however, the CB and PT do not provide the proper floor structure for the measurement to be performed in the order in which the protocol recommends [9]. For this reason, lameness was assessed when the cows left the milking parlor. This adaptation of the WQ protocol was based on the considerations of Veissier et al. [10] (pp. 115–146), who suggested that one of the probable adaptations for the inclusion of the grazing system in the evaluation of the WQ protocol would consist of carrying out the lameness evaluation when the cows were taken for milking. This is justified by the more uniform floor and the proximity of cows, which this location allows the observer.

### 2.3. Welfare Quality^®^ Protocol Measure Score

To define the overall AW of the properties, according to the WQ protocol, it was necessary first to collect the specific scores of the measures referring to the 11 criteria, and then to collect the scores of the criteria referring to the four principles and, finally, to join the scores obtained on each of the four principles to obtain the global AW score for each property [9].

The combination of the scores of the measures of a criterion formed the final score of this. The score obtained from the combination of these measures varies from 0 to 100. Zero refers to the worst level of AW (low grade) and 100 to the best level (high grade). With the combination of the scores obtained in the 11 criteria, it was possible to form the score of the four principles on a scale from 0 to 100 as well. Furthermore, the combination of the scores obtained in the four principles resulted in the global assessment of the property (0 to 100) [9].

### 2.4. Selection of Variables for Statistical Analysis

The criteria “Expression of Other Behaviors” (OTHERS) and “Ease of Movement” were excluded from the comparative analysis (Table 1) since the score of the OTHERS criterion is given for farms that allow access to pasture and none of the evaluated feedlots allowed, resulting in scores of maximum values only for the PT. The criterion “Ease of Movement” obtained maximum score in the systems, with no significant difference between them, as the cows of the evaluated farms did not remain tied, as is observed in the management of the tie-stall system.

In the analysis of the measures, we used the values that the WQ protocol program [22] requires to perform the calculation to obtain the classification of dairy farms. The measures selected for analysis were only those describing the criteria that showed a significant difference in the comparative statistical analysis between the systems (Table 1).

### 2.5. Statistical Analysis

Initially, descriptive statistics were calculated (mean, standard error of the mean, median, minimum, maximum, and quartiles). Box-plot graphs were designed to assess data dispersion according to the factors evaluated.

To compare the data obtained in the different milk production systems, the Kruskal–Wallis non-parametric method was used due to the variables not having a normal distribution or not having met the assumptions of an analysis of variance. The justification for using the median as a comparison parameter is due to the characteristic of non-parametric methods, as it was considered a realistic and viable value that farmers could aspire to with the objective of improving their classification. Additionally, the chi-square test was performed to measure the water flow and cleanliness of water points, whose significance was attested when the p-value was less than 0.05. For all analyses, the statistical software R [23] was used. A similar method of statistical evaluation was used in the work of De Vries et al. [24].

### 2.6. Ethics Committee

The Ethics Committee (Ceua/UDESC) approved the experiment, under protocol Ceua nº 7148040719–ID 00997.

## 3. Results

The analysis of the 51 dairy farms was carried out to compare the three systems about measures, criteria, and principles of the WQ protocol. The AW measures analyzed were those related to the criteria that showed statistical differences between the systems (in bold in Table 1), i.e., measures that made up the criteria “Absence of Prolonged Thirst” (APT), “Comfort in the Rest Area” (CRA), “Absence of Injury” (AI), “Absence of Disease” (AD), “Good Human–Animal Relationship” (HA) and “Positive Emotional State” (PES).

### 3.1. Overall Assessment

In the global assessment of AW based on WQ protocol, 10 FS farms were considered “Acceptable” and seven “Unclassified”. In the CB farms, 13 were considered “Acceptable” and four “Not Classified”. In the PT, 10 farms were classified as “Enhanced”, one “Acceptable” and six as “Unclassified”.

### 3.2. Analysis of the Principles

The analysis of the principles (Table 2) showed that “Good Feeding” did not show any significant difference between the systems. In the “Good Health” and “Appropriate Behavior” principles, the PT presented better scores compared to confinement systems, moreover FS did not differ statistically from CB. In the “Good Housing” principle, the PT had the best score, followed by CB and FS, the latter with the worst score.

The farms of the CB and FS systems did not score below the decimal level in the APT criterion and the “Good Nutrition” principle, with only three PT receiving the maximum score in this criterion (“Excellent” classification), while in the CB there were 10 (58.8%) and in the FS, 11 (64.7%).

### 3.3. Analysis of Measures and Criteria

The median values of the number and total length of the drinkers (APT criterion) were significantly lower for the PT concerning the confinement systems, indicating the weakness of the PT in this area (Table 1). On the other hand, the systems did not show differences concerning the flow and cleanliness of the water points (Table 1).

In the analysis of the CRA criterion, cows in the FS took longer to perform the necessary movements to lie down than cows in the other two systems (Table 1). The FS also showed higher collision measures with installation equipment at bedtime and cows lying outside the rest area. In assessing body cleanliness (CRA criterion), the CB differed from the other two systems, presenting a higher percentage of cows with dirty lower hind legs. Furthermore, the PT had a lower percentage of dirty cows than the two confinement systems, with the FS and CB not differing in cows with dirty udders and flanks.

In the measures belonging to the AI criterion (Table 1), the PT presented a higher percentage of cows without integument alternations when compared to the confinement systems and did not differ from the FS in the measurement of mild integument alternations; however, it was higher than the CB. In addition, the PT presented lower medians concerning the confinement systems in the assessment of severe alternations, indicating a lower number of injured cows than the confinement systems, which did not differ from each other in this measure. The PT stood out positively in the absence of lameness, as it presented a higher median concerning the CB, which, in turn, had a higher median value concerning the FS (Table 1). Severe lameness (score 2) was observed in many cows in the FS compared to PT, and the CB did not differ from both (Table 1). The descriptive analysis of the data shows that the incidence of cows with severe lameness was 8.05% in the FS, while the CB presented 4.21%, and the PT, 1.37%.

In the AD (Table 1), the measures that showed a significant difference were those of nasal, ocular, and vulvar discharge, diarrhoea, and mortality. Vulvar discharge and mortality were prevalent among cows evaluated in the FS. The confinement systems did not differ from one another in the medians of nasal and ocular discharge and differed from the PT, which had less affected cows. The measured qualitative assessment of behavior (PES criterion) (Table 1) showed variation between systems and emotional states that refer to positive feelings (such as happy, excited, relaxed, positively occupied, and calm), which had a higher incidence in PT herds than that in confined herds. In contrast, the emotional states that indicate negative feelings (such as bored, distressed, frustrated, and fearful) were prevalent in the confinement systems, with no significant difference.

In the HA criterion, the occurrence of score 1 of the distance avoidance measure (cows that allowed to be touched) was not different between the systems evaluated (Table 1). However, the PT presented more cows classified in score 2 (approximation less than 50 cm) compared to CB and FS systems and fewer cows that did not allow to be touched at a distance less than 1 m (score 4) than those confinement systems, with a worse performance of the FS, indicating a higher occurrence of reactive cows.

The comparison in terms of criteria of the WQ protocol indicates that there was no difference between the systems in the criteria “Absence of Prolonged Hunger” (APH), “Absence of Pain Induced by Management Procedures” (PAIN), and “Social Behaviors” (SB) (Table 1).

The PT did not differ from the CB in the AD criterion, but it presented a better result when compared to the FS. On the other hand, the CB and FS systems did not differ in the AD criterion (Table 1). In the APT criterion, the PT had lower medians than both confinements (Table 1). In the other criteria in which there were significant differences between systems (CRA, AI, HA, and PES), the PT presented the highest median values, indicating better scores in the assessment of the welfare of dairy cows, followed by the two feedlot systems that had an equal performance in the AI, HA, and PES criteria. However, in the CRA criterion, the FS presented worse conditions than the PT and CB (Table 1).

**Figure 1:**
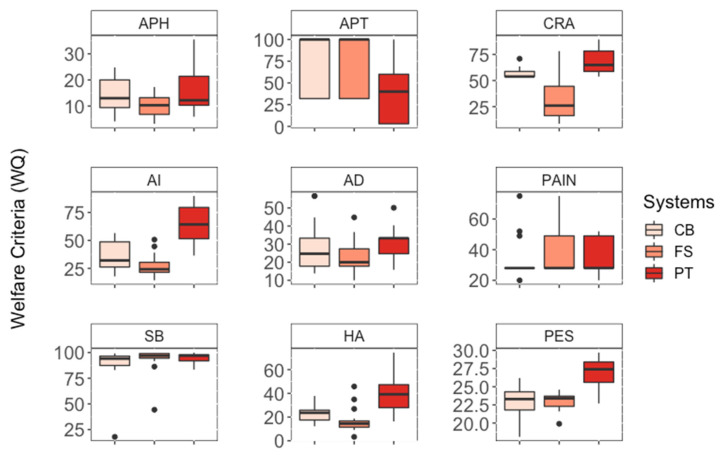
Box-plot of the Welfare Quality^®^ (**WQ**) protocol criteria: “Absence of Prolonged Hunger” (**APH**), “Absence of Prolonged Thirst” (**APT**), “Comfort in the Rest Area” (**CRA**), “Absences of Injuries” (**AI**), “Absence of Diseases” (**AD**), “Absence of Pain Induced by Management Procedures” (**PAIN**), “Social Behaviors” (**SB**), “Good Human–Animal Relationship” (**HA**) and “Positive Emotional State” (**PES**), in compost barn (**CB**), free stall (**FS**) and pasture-based (**PT**) management systems.

In the analysis of the criteria by the box-plot (Figure 1), the greatest dispersion of the data in the APT criterion stands out, showing great variation between the farms evaluated in the FS, CB, and PT. In this criterion, PT presented the lowest scores in comparison to the confinement systems. For APH, PAIN, and SB (very high, close to 100), there is little distance between the medians, indicating a possible absence of difference between treatments. In the others, there is a high score for the pasture-based system in all of them (Figure 1).

## 4. Discussion

The analysis of the WQ protocol showed that the main problem with PT was in relation to the adequate supply of drinking water (criterion APT) (Table 1), which was also observed in another experiment in Brazil [25]. This lack can affect water consumption [26,27], milk production [28], and health [29]. In addition, herds in pasture with free access to drinking water presented 10% more in milk production [30].

In our study, the PT farms that did not have water points in the paddocks, allowed the water intake only twice a day, at the exit of the milking parlor. This aspect characterizes an AW problem, because the average frequency/day for water intake can be 7.3 (± 2.8) for dairy cows [31], which suggests that the possibility of drinking water only twice a day is insufficient.

Beggs et al. [32] used a questionnaire about the type of supply, time of access, and location of water points of 50 PT dairy herds. Although these questions add essential information, studies show that cows avoid drinking manure-contaminated water [33], so we considered it important to keep the visual assessment as recommended by the WQ protocol. 

In the APH criterion, there were no significant differences between the systems. However, the medians of the values were low for all (Table 1), suggesting that dairy cows are maintained at below ideal nutrition scores and those found in the other literature [34,35]. Popescu et al. [34] found APH criterion values of 58.9 and 52.5 for cows kept in tie-stall properties with or without access to the external area, respectively. In FS with rubber mat beds, the average was 41.4, and with deep straw beds the average was 49.6, in Lower Saxony, Germany [35]. In confined systems with ≥12 h of access to pasture/day, the average score was 71 in winter and 53 in summer, in various climate zones and soil zones in Germany [36]. In our work, the score of the union of the three systems evaluated in the APH criterion was 13.0, which is about 76.5% lower than the mean of the values in the cited literature (average of 55.5), showing that this aspect is a critical point of AW in the region studied.

The low scores of body score of the cows evaluated in this study (Table 1) may favor the appearance of lesions and lameness, as was also demonstrated in the studies by Randall et al. [37] and Oehm et al. [38]. Furthermore, the system that had the worst scores concerning lameness was the FS. Cows with higher body scores are less injured when lying down and standing up, as the prominence of bones and the fragility of hair due to poor nutrition can favor the appearance of alopecia and injuries [39] (pp. 43–50). In addition, injured or claudicating cows tend to have a lower body score to reduce the weight on the affected limb, considering that pain reduces food intake [40].

Our results showed that CB and PT were superior to FS in relation to comfort, such as lying behavior (criterion CRA) (Table 1). Cows evaluated in FS in Germany presented duration of movements for lying down from 5.36.0 s [41], which are values close to the FS in our work (5.2 s) (Table 1). It is possible that the longer time required to lie down in FS was affected by the smaller number of non-lame cows in this system (criterion AI) (Table 1) and what was also evidenced by Popescu et al. [34]. Cows injured or in pain processes are more prone to collision when they go to bed and have more inappropriate positions of decubitus outside the resting area [42]. If bed and cubicle dimensions are adequate, it will result in fewer injuries and less hesitant behavior before bedtime [43].

The number of collisions in our study (median 26.7%) for the FS (Table 1) was higher than that founded in Germany by Gieseke et al. (average 17.9%) [42] and in the United Kingdom by Heat et al. (median 20%) [44]. A recent work showed a positive correlation between the duration of the movements required to lie down and the percentage of collisions; and between the frequency of cows lying outside the resting area with severe integument alternations [45]. Furthermore, there was a higher percentage of collisions when the cows had higher levels of mastitis and lameness, as pain is the potential to reduce the mobility of cows [45]. The surface of the resting area also affects comfort and cows in FS avoided lying on wet and dirty surfaces, decreasing behavior up to 5 h daily [46]. 

Like our results of body cleanliness (Table 1), a lower prevalence of integument alternations and greater in the dirt was found in cows reared in CB than the FS [21]. On the other hand, it is essential to consider that management can influence the bedding quality and the dirt score of cows reared in CB [13,47] and, in some cases, may not occur differences in the dirt score between FS and CB [48].

In our work, the PT presented better scores in relation to the criteria associated with comfort and health. Similar results were observed in a study carried out in 61 German loose housing dairy farms [35]. Armbrecht et al. [35] demonstrated that confinement systems that enabled access to pasture obtained better scores in the CRA, AD, and AI criteria, compared to farms that did not allow access. Likewise, in an experiment carried out in Costa Rica (with intensive, semi-intensive, and extensive systems), the “Good Housing” principle showed higher values for systems in which cows spend part or all day on pasture [15]. 

The pasture-based management system of our work was the one that presented a better condition in CRA and AI criteria (Table 1). In agreement with our results, studies have demonstrated that cows are less injured in places with more comfortable resting areas [49]. The lameness was considered as the most important problem in intensive systems of dairy farming, concerning AW, health, and milk production [49]. Lameness and interdigital dermatitis were associated with greater dirtiness in the locomotor limbs [40]. Furthermore, a higher percentage of lameness was related to worse comfort conditions, such as slippery floors due to excessive humidity, manure and mud, and hock injuries in FS [50].

In the score 0 of the lameness measure, the CB was better than the FS in our work (Table 1). In agreement, other studies have found that cows housed in the CB had fewer hock injuries and swelling compared to the FS [48] and a lower occurrence of lameness [48,51]. However, another study carried out in Girona (Spain) with 575 cows, resulted in a higher proportion of severely lame cows in the CB than in the FS [21]. The differences found in the works can be explained by several factors related to management practices, cows, and the characteristics of the facilities [51].

Lobeck et al. [52] demonstrated a higher occurrence of lameness in the FS concerning the CB. Other studies that compared these confinement systems showed a higher frequency of severe integument alternations [21], foot problems, such as erosion of the heel, chronic laminitis, and interdigital hyperplasia [53] at the FS system. In addition, a lower prevalence of hock injuries, reproductive diseases, and mastitis [54] (pp. 119–151), and a decrease in the occurrence of lameness [55] were related to pasture. Pasture floor is considered the gold standard concerning mobility comfort when compared to other floors, such as asphalt and solid rubber [56]. 

However, in the “Good Health” principle, the clinical symptom that had the highest incidence was nasal discharge (Table 1). This symptom affected up to 71.4% of the cows evaluated in one of the evaluated FS properties. Despite the greater risk of contracting infectious diseases of the respiratory tract, such as bovine herpes virus, pasture systems have a lower density of cows in a closed place, making it difficult to spread this and other diseases compared to confinement systems [57]. Molina et al. [45] correlated respiratory problems with cleanliness, lower estrus detection rates and lower milk production.

The FS had worse scores in the “Good Housing” principle (Table 2) and was worse than the PT in the criterion AD (Table 1), which may have cooperated for a higher mortality rate, because it is associated with health problems [58]. The under occurrence in the PT did not differ from other studies that observed a decrease in mortality in farms that allowed access to pasture [59], which may show a decrease of up to 75% in mortality [60]. In addition, herds of 50 to more than 100 cows presented a high risk of mortality compared to smaller herds, from 35 to 50 cows [59]. 

The evaluation of diarrhoea in the PT did not differ from that observed in the FS and CB (Table 1). Unlike our results, Burow et al. [60,61] found a higher occurrence of diarrhoea in confinement with access to pasture. This could be explained due to the excess of protein and sucrose from fresh forage present in pastures in the hottest times of the year [36].

As for the greater number of cows with vulvar discharge found in FS (Table 1), Grimard et al. [62] found positive associations between good reproductive performance of cows and AW, and Molina et al. [45] found that inadequate body scores and a longer duration of movements required for bedtime were positively correlated with a calving-first insemination interval, which indicates that reproductive problems may be related to worse comfort conditions in the rest area. These aspects were worse scored by the FS in our work (Table 1). In addition, Arnott et al. [58] suggest benefits of the pasture system concerning reproductive aspects, which was evidenced by Bruun et al. [63] when associating a lower risk of developing metritis when cows have access to pasture.

The PT showed better results in HA (Table 1). This result differs from that found by Battini et al. [64], in which the cows had a greater avoidance distance soon after being confined in a loose housing system, after spending four months on pasture. The authors suggested that cows began to trust humans less after this time since in pastures, as there is less frequency of human–animal interaction than in confinement [64].

In contrast and similarly to our results, the work of Aigueperse [65] demonstrated that dairy cows with access to pasture showed lower reactivity than cows kept all the time in confinement. It is worth noting that positive interactions with humans will result in a shorter avoidance distance. Studies indicate that negative attitudes of producers toward cows result in low scores in the assessment of AW, and that positive attitudes were correlated with high scores in the “Appropriate Behavior” principle [66]. The quantity, quality, and continuity of the contact and handling of cows are the variables that most affect the evaluation of the human–animal relationship [67], and keepers with aversive behaviors can cause fear in cows, increasing up to 70% the residual milk [68].

The highest medians evidenced the best evaluation of PT in the PES criterion in positive characteristics, such as relaxed and happy, and lowest medians in negative ones, such as fearful and frustrated (Table 1). The association between positive emotions and permanence in pasture was also demonstrated by Motupalli et al. [69] because cows had a preference to spend more time on pasture (68.7% of the day) than in FS. Although animal preferences are not directly related to better AW [70], they are related to a more positive emotional state. Cows kept on pasture can spend up to 68% of the daily time in feeding behaviors, however when cows are kept in confinement, only 22% of the daily time is devoted to this behavioral category [14], differing from the natural behavior [71], favoring temporal leisure and negative emotional states [72]. 

However, the opportunity for choice may not necessarily relate to improved AW, as animals may not choose what is in the best interest of their welfare. Access to pasture was related to positive emotional states by assessing the cows’ body expressions, such as sclera of the eyes and ear positions [73], as well as with the criteria AD and SB in addition to that of the PES [36]. The PT allows a greater expression of motivated behaviors and, consequently, a decrease of boredom [72].

## 5. Conclusions

This work demonstrates that the production system affects the welfare of dairy herds and that the pasture-based system presents better scores in the global welfare assessment and in most of the measures evaluated, with the main exception the measure related to an adequate water supply. The free stall system has more critical points of well-being than the others two systems, with the compost barn system having some scores similar to the pasture-based system, and others to the free stall, depending on the measure evaluated. However, it has advantages in the principle “Good Housing”, when compared to the free stall system. The critical points of the animal welfare of each system must be carefully considered to make the necessary changes to increase the degree of well-being and quality of life of dairy cows.

## Figures and Tables

**Table 1:** Scores of the criteria and measures of the Welfare Quality^®^ protocol in the compost barn (CB), free stall (FS) and pasture-based (PT) systems in Santa Catarina, Brazil.

Criteria * and Measures	Milk Production Systems				Overall *p*-Value
	CB (*n* = 17)	FS (*n* = 17)	PT (*n* = 17)	CB+FS+PT (*n* = 51)	
Absence of Prolonged Hunger *	13.0	10.3	12.2	13.0	0.1275
Absence of Prolonged Thirst *	**100 ^a^**	**100 ^a^**	**40.0 ^b^**	**60.0**	**0.0234**
Number of water points	4.0 ^a^	5.0 ^a^	1.0 ^b^		<0.001
Total length of troughs (cm)	760.0 ^a^	1000.0 ^a^	120.0 ^b^		<0.001
Water flow					0.9913
0—No	0.06	0.00	0.14		
1—Yes	0.94	0.94	0.86		
2—Partially	0.00	0.06	0.00		
Cleanliness of water points					0.8860
0—Dirty	0.35	0.29	0.07		
1—Clean or partly dirty	0.65	0.71	0.93		
Comfort in the Rest Area *	**53.8 ^b^**	**26.0 ^c^**	**64.9 ^a^**	**53.8**	**<0.001**
Time needed to lie down (average)	3.6 ^a^	5.2 ^b^	3.3 ^a^		<0.001
Collision during lying down (%)	0.0 ^a^	26.7 ^b^	0.0 ^a^		<0.001
Animals lying outside the lying area (%)	0.0 ^a^	18.9^b^	0.0^a^		<0.001
Cleanliness (%)					
1—Cows with dirty udder	93.3 ^b^	88.6 ^b^	78.9 ^a^		0.0249
2—Cows with dirty hindquarters	28.6 ^b^	25.0 ^b^	13.3 ^a^		0.0015
3—Cows with dirty lower hind legs	33.3 ^b^	15.6 ^a^	9.7 ^a^		0.0037
Absence of Injuries *					
Integument alterations					
0 – No integument alterations	43.3 ^b^	25.6 ^b^	62.5 ^a^		<0.001
1 – Mild integument alterations	0.0 ^a^	10.0 ^b^	12.5 ^b^		0.0271
2 – Severe integument alterations	50.0 ^b^	59.4 ^b^	20.7 ^a^		<0.001
Lameness (%)					
0—Not lame animals	90.0 ^b^	85.7 ^c^	100.0 ^a^		<0.001
1—Lame	3.3 ^ab^	8.6 ^b^	0.0 ^a^		<0.001
2—Severely lame	3.3 ^ab^	6.7 ^b^	0.0 ^a^		<0.001
Absence of Diseases *	**24.7 ^ab^**	**20.0 ^b^**	**33.3 ^a^**	**24.7**	**0.0332**
Coughing (%)	0.05	0.05	0.03		0.4517
Nasal discharge (%)	46.7 ^b^	51.9 ^b^	18.8 ^a^		0.0013
Ocular discharge (%)	6.7 ^b^	6.7 ^b^	0.0 ^a^		0.0496
Vulvar discharge (%)	0.0 ^a^	2.7 ^b^	0.0 ^a^		0.0496
Hampered respiration (%)	0.0	0.0	0.0		0.0694
Diarrhoea (%)	6.7 ^a^	20.0 ^b^	13.9 ^ab^		0.0167
Milk somatic cell count (%)	0.0	0.0	0.0		0.2771
Downer cows (%)	2.9	4.2	4.3		0.3896
Mortality (%)	6.5 ^ab^	9.7 ^c^	3.1 ^a^		0.0046
Dystocia (%)	2.2	3.7	5.0		0.6150
Absence of Pain *	28.0	28.0	28.0	28.0	0.2454
Social Behaviors *	94.1	96.9	97.0	96.0	0.1595
Good Human–Animal Relationship *	**23.6 ^b^**	**14.5 ^b^**	**39.2 ^a^**	**23.6**	**<0.001**
Avoidance distance (%)					
1—Animals that can be touched	13.3	11.4	15.6		0.3260
2—Approximation less than 50 cm	16.3 ^b^	8.1 ^b^	34.4 ^a^		<0.001
3—Approximation between 100 and 50 cm	21.9 ^a^	14.3 ^a^	23.1 ^a^		0.0436
4—Approximation greater than 100 cm	40.0 ^b^	67.4 ^c^	13.3 ^a^		<0.001
Positive Emotional State *	23.3 ^b^	23.4 ^b^	27.4 ^a^	23.8	<0.001
Tendency to be active	7.5 ^ab^	6.5 ^b^	8.5 ^a^		0.0116
Tendency to be relaxed	4.5 ^b^	3.1 ^b^	9.4 ^a^		<0.001
Tendency to be fearful	6.0 ^b^	7.5 ^b^	2.5 ^a^		0.0017
Tendency to be agitated	3.5 ^b^	3.0 ^ab^	2.0 ^a^		0.0436
Tendency to be calm	3.6 ^b^	4.3 ^b^	10.0 ^a^		<0.001
Tendency to be content	2.2 ^b^	2.0 ^b^	9.0 ^a^		<0.001
Tendency to be indifferent	1.6	2.2	1.9		0.6892
Tendency to be frustrated	2.3 ^b^	3.5 ^b^	0.0 ^a^		<0.001
Tendency to be friendly	3.1	5.4	6.2		0.2786
Tendency to get bored	2.5 ^b^	4.0 ^b^	0.0 ^a^		<0.001
Tendency to be playful	0.0	0.0	0.0		0.1286
Tendency to be positively occupied	4.9 ^b^	7.0 ^b^	9.0 ^a^		0.0028
Tendency to be lively	0.8 ^b^	0.0 ^b^	5.5 ^a^		<0.001
Tendency to be inquisitive	2.0	1.9	2.4		0.2939
Tendency to be irritable	2.3 ^b^	1.5 ^ab^	0.5 ^a^		0.0105
Tendency to be uneasy	2.3 ^b^	2.0 ^ab^	0.0 ^a^		0.0127
Tendency to be sociable	7.6 ^a^	8.0 ^a^	8.5 ^a^		0.0359
Tendency to be apathetic	2.0 ^b^	2.0 ^ab^	0.0 ^a^		0.0246
Tendency to be happy	0.0 ^b^	0.5 ^b^	5.3 ^a^		<0.001
Tendency to be distressed	2.6 ^b^	3.5 ^b^	0.0 ^a^		<0.001

^a–c^ Medians with different envelopes in the lines differ between milk production systems (*p* < 0.05). In bold: criteria that showed statistical differences between the compost barn, free stall and pasture-based systems. * Criteria.

**Table 2:** Scores of principles of the Welfare Quality^®^ protocol in the compost barn (CB), free stall (FS) and pasture-based (PT) systems in Santa Catarina, Brazil.

Principles	Milk Production Systems			CB + FS + PT (*n* = 51)	Overall *p*-Value
	CB (*n* = 17)	FS (*n* = 17)	PT (*n* = 17)		
Good Feeding	32.2	31.4	26.6	30.0	0.1153
Good Housing	70.9 ^b^	53.4 ^c^	77.9 ^a^	70.9	<0.001
Good Health	24.4 ^b^	22.8 ^b^	34.0 ^a^	27.1	<0.001
Appropriate Behavior	18.3 ^b^	16.0 ^b^	37.5 ^a^	18.8	<0.001

^a–c^ Medians with different envelopes in the lines differ between milk production systems (*p* < 0.05). Scores of principles from 0 to 100.

## Data Availability

The data presented in this study are available on request from the corresponding author. The data are not publicly available due to personal reasons.
